# Letting Go of Self: The Creation of the Nonattachment to Self Scale

**DOI:** 10.3389/fpsyg.2018.02544

**Published:** 2018-12-13

**Authors:** Richard Whitehead, Glen Bates, Brad Elphinstone, Yan Yang, Greg Murray

**Affiliations:** Department of Psychological Sciences, Swinburne University of Technology, Melbourne, VIC, Australia

**Keywords:** nonattachment, nonattachment to self, scale development, self-concept, Buddhist psychology

## Abstract

The Buddhist notion of nonattachment relates to an engagement with experience with flexibility and without fixation on achieving specified outcomes. The present study sought to define, create and validate a new measure of nonattachment as it applies to notions of the self. A new construct of “nonattachment to self” (NTS) was developed, defined the absence of fixation on self-related concepts, thoughts and feelings, and a capacity to flexibly interact with these concepts, thoughts and feelings without trying to control them. Two studies were conducted in the development of the new scale. With expert consultation, study 1 (*n* = 445) established a single factor, internally consistent 7-item scale via exploratory factor analysis. Study 2 (*n* = 388, *n* = 338) confirmed the factor structure of the new 7-item scale using confirmatory factor analyses. Study 2 also found the new scale to be internally consistent, with evidence supporting its test-retest reliability, criterion, and construct validity. Nonattachment to self-emerged as a unique way of relating to the self, distinct from general nonattachment, that aligned with higher levels of well-being and adaptive functioning.

## Introduction

A person's notion of self has become an important element in research on individual suffering. The sense of self, and fixations on self-focused thoughts and feelings are associated with a range of negative psychological symptoms such as depression and anxiety (Lemogne et al., 2009; Kyrios, 2016). Recently, concepts from Buddhist psychology (i.e., understanding the Buddhist study of the human condition though current psychological knowledge; Olendzki, 2003) have been investigated in relation to a negative relationship with self. Interventions based on self-compassion and mindfulness that positively address how individuals relate to their self, have been associated with a range of positive psychological outcomes (Shonin et al., 2014; Wayment et al., 2014; Woodruff et al., 2014). Nonattachment is another Buddhist construct that has recently been shown to have major psychological benefits (Tran et al., 2014; Ju and Lee, 2015; Sahdra et al., 2016) but is yet to be investigated in relation to the self.

Nonattachment directly captures an individual's relationship with their experience and highlights a capacity to suspend attempts to control experience through clinging to experiences perceived as desirable or avoiding experiences perceived as undesirable (Sahdra et al, 2010; Sahdra et al., 2016). An important dimension of nonattachment that is central to the Eastern contemplative traditions, is nonattachment to an independent, static self (Rāhula, 1959; Hanh, 1998; Hanson, 2009; Thubten, 2009). Although a measure has been developed to assess nonattachment in terms of how it relates to one's life in general (Sahdra et al, 2010), currently there is no measure that directly assesses nonattachment in relation to the self. The present study aimed therefore, to develop a measure of “nonattachment to self,” conceptualized as the extent to which individuals can interact with their self-related concepts, thoughts and feelings without fixation, and without a need for the self to be different than it is.

### The Role of the Self in Suffering

The way we perceive and interact with “self” is an important determinant of our behavior and quality of life. While there is no agreed framework in psychology for researching the important folk idea of “self,” there is a resurgence of interest in self-related constructs, especially in clinical psychology (Kyrios et al., 2016). One theme in the current literature posits that many facets of well-being are negatively impacted by an intrapersonal stance which elevates the self-concept as a fixed thing through which experience is filtered and weighed. Perceptions of this fixed self-concept which are overly negative have shown to relate to negative mental health symptoms such as anxiety and depression (Beck et al., 1989; Mor and Winquist, 2002; Lemogne et al., 2009), whereas fixating on positive self-concepts can be associated with narcissism, excessive defensiveness (Rhodewalt and Eddings, 2002) or feelings of superiority over others (Egan, 1997).

In Buddhism, a self that exists independent of experience is seen as illusory, and it is considered a delusion to believe that happiness arises out of fulfilling the desires of such a permanent self (Scarborough, 2009). Ignorance is also defined by the grasping at the separate self, in which power is given to the perceived existence of a self that is the ruler of experience (Dalai Lama, 2009). This mistaken perception drives people's attempts to protect the self-delusion causing anxiety and suffering (Chang et al., 2014). In the Buddhist psychological literature, it is this identification of the self as fixed, and the fixation on either positive or negative aspects of self, that can be defined as attachments toward the self. Theoretically, it is attachment to the self that creates egoic functioning (Ardelt, 2008; Van Gordon et al., 2016) and thus lies at the core of individual suffering (Hanh, 1998; Dalai Lama, 2001). The Buddhist path involves a drive toward letting go of this attachment to the static self (Donner, 2010) and thus a transcendence of personal suffering.

Attachments to the self can emerge in many forms. The construct of inner defenses, or defense mechanisms highlight attachments to the self. In theory, such defenses aim to preserve the self-concept by keeping away anything perceived to be incongruent with the self-structure, even if this is detrimental to the self (Rogers, 1965; Kernis and Heppner, 2008). For example, if an individual receives criticism they perceive as a threat to self-esteem, they can engage in defenses such as dismissing the experience or the person communicating it, as a means to protect their self-esteem and view of self. Similarly, experiences that underlie a vulnerability to depression such as excessive shame or guilt (Kim et al., 2011) can also be viewed as attachments toward a static, unchanging self (Whitehead et al. submitted) and arise when the self is harshly judged or is judged to be fundamentally flawed (Kyrios et al., 2016).

Many psychological interventions address factors associated with the self-concept that exacerbate negative psychological symptoms (Kyrios et al., 2016). For example, schema therapy aims to draw attention to maladaptive schemas about the self and seeks to heal unhelpful schemas and build healthier responses to experience (Rafaeli et al., 2016). Similarly, cognitive behavioral therapy aims to produce therapeutic change by modifying individuals' biased and unhelpful self-representations (Clark, 2016). More recently, mindful self-compassion interventions have been shown to reduce the impact of depressive symptoms (Pauley and McPherson, 2010; Krieger et al., 2013; Friis et al., 2016) through building a kinder, accepting and more compassionate relationship to self (Neff, 2008).

Self-compassion is a further construct rooted in Buddhist psychology, and research indicates that taking a more self-compassionate, balanced stance toward the self can be beneficial for mental health (e.g., Neff, 2003). Self-compassion involves a non-attached position toward negative self-focus and “requires taking a balanced approach to one's negative emotions so that feelings are neither suppressed nor exaggerated” (Neff, 2008, p. 98). Like nonattachment to self, self-compassion incorporates the benefits of taking a less rigid approach to self. One difference between self-compassion and nonattachment to self is that self-compassion emphasizes overcoming negative self-focus, whereas nonattachment to self involves removal of an over-focus on the self, regardless of valence. In theory, any attachment or fixation on the self-concept, whether good or bad, can be problematic due to the ever-changing nature of experience. For example, if an individual clings to positive notions of self, such as being a “good student,” if this positive view is challenged by receiving a bad mark on an exam, this can elicit feelings of defensiveness, putting others down, or further attempts to compensate for the incongruence between that ideal self-concept and the reality of the situation which is ever-changing (Epstein, 2007). Being non-attached toward the self, therefore, limits incongruence between experience and the self-concept, allowing an individual to move through their life with greater flexibility, an understanding of the ever-changing nature of the self and a view of self that is free from expectation and fixation.

In addition to Buddhist conceptualizations, the notion of being non-attached toward the self also appears to be a key theme in the optimal stages of psychological health (Ardelt, 2008). Moving beyond self-fixation and the concerns of the individual self is a core component of a range of theories of optimal psychological functioning. Rogers (1961) and Maslow (1954) both proposed that individuals operating at the higher stages of psychological development demonstrate a reduced fixation on the self and a propensity to move beyond self-interest toward a more other- and universal-focus. Similarly, theories of adult development such as Levenson et al.'s (2001) liberative model of adult development or Loevinger's (1976) stages of ego development propose the higher stages of adult development involve a reduction of attachment toward the ego and a transcendence of self-focus and self-fixation.

Although the benefits of nonattachment to the self have been outlined in theory, no research has been conducted on the construct of nonattachment to self. In the absence of any established measure of nonattachment to self, research on the more general construct of nonattachment shows that letting go of attachments and attempts to control experience in general, is beneficial for well-being. Research using Sahdra et al's (2010) nonattachment scale (NAS) shows that higher levels of nonattachment are associated with greater short-term, subjective well-being (Sahdra et al, 2010), more longer-term, pervasive psychological well-being, (Ju and Lee, 2015; Whitehead et al., 2018), and reduced amounts of negative psychological symptoms such as rumination (Coffey and Hartman, 2008), depression, anxiety, and stress (Sahdra et al, 2010). These findings suggest the energy spent trying to cling to or avoid experience can inhibit a greater sense of presence and well-being across a range of different areas in a person's life (Sahdra et al, 2010), and that letting go of attachments can ameliorate the impact of negative mental health symptoms.

### The Present Research

The present research involved two sequential studies directed at creating a psychometrically valid measure of nonattachment to self. As general nonattachment appears to have psychological benefits, nonattachment specific to the self may be equally, or more beneficial. Study 1 details the development of a scale to measure nonattachment to self in the general population. This involved an initial consultation with primary and secondary texts as well as consultations with experts in the field to develop an item pool. These items were then subjected to exploratory factor analysis (EFA), and internal consistency of items in identified factors was established. Study 2 examined the validity of the factors identified in Study 1 via two confirmatory factor analyses (CFAs). The new scale was also tested for internal consistency and test-retest reliability, as well as criterion, convergent and discriminant validity. Furthermore, establishing a nonattachment to self measure that is distinct from general nonattachment was crucial to the validity of the new measure. Therefore, a discriminant analysis using nested models in CFA was conducted to test the distinctiveness of the new scale.

## Study 1: Scale Development and Content Validation

### Preliminary Item Construction

The first stage of scale development involved creation of an initial item pool. Numerous primary texts were consulted (two of the major text consulted were the Abidhamma, 1993, third century BCE and the Upanishads, 2000, 8–5th BCE) as well as numerous contemporary texts from Eastern contemplative traditions that address notions of no-self and nonattachment to self (some of the major texts consulted were: Hanh, 1998, 2006; Thubten, 2009; Adyashanti, 2012). A total of 30 items was developed from this research. The second stage involved a two-step consultation process with seven experienced teachers and practitioners from relevant disciplines (i.e., Theravadin Buddhism, Mahayana Buddhism, Adavita/Vedanta). These experts were experienced in theory and practice relating to ego-attachment and letting go of attachment to the egoic self. This consultation helped define the construct and the item pool was increased based on this definition. As the existing measure of nonattachment is a reliable and well-validated measure (e.g., Arch et al., 2016; Sahdra et al., 2016; Van Gordon et al., 2016). Sahdra et al's (2010) definition of nonattachment was used in consulting with experts. Nonattachment was defined as the “subjective quality of not being stuck or fixated on ideas, images, or sensory objects and not feeling an internal pressure to acquire, hold, avoid, or change” (Sahdra et al, 2010, p. 118).

The first step of the consultation process produced a definition of nonattachment to individuals' self-related thoughts, feelings, and concepts. Nonattachment to self was defined as the absence of fixation on self-related concepts, thoughts, and feelings and a capacity to flexibly interact with these concepts, thoughts, and feelings without trying to control them. On the basis of suggestions given by the experts and insights gained during discussion, 86 new items were created, resulting in a total item pool of 116 for further investigation. The number of items in the item pool are in line with previous research into similar scales measuring nonattachment (Sahdra et al, 2010) and mindfulness (Brown and Ryan, 2003).

In the second step of the consultation process, experts rated the 116 items on clarity and the extent to which each item captured the construct. A number of items were found to lack clarity or failed to capture the agreed upon nature of the construct. Other items were identified as lacking appropriateness for non-meditators, or for inadvertently assessing related but distinct constructs (e.g., mindfulness, self-transcendence). The process of consulting relevant texts and experts highlighted that in Buddhism, nonattachment to self is discussed in terms of developing an understanding of the illusory nature of the self. However, as the construct of nonattachment to self-needed to be applicable to the general population, with or without meditation experience, most items referring to the non-existence of a separate self, or illusory nature of the self were removed. On completion of the review stage of the consultation process, 64 items remained for exploratory factor analysis (EFA).

### Method

#### Participants

The sample of 445 comprised 124 men and 321 women who aged from 18 to 77 years (*M* = 35.77, *SD* = 11.84). Most respondents did not report any religious or spiritual affiliation (51.2%), others identified as Christian (22.2%); 10.8% identified with a general, non-religious spirituality, 8.3% identified with a contemplative tradition (i.e., Buddhism, Vedanta), 2.7% identified as Muslim, 1% identified as Hindu and 3.8% other. The majority of participants (51.7%) engaged with a contemplative practice (e.g., meditation, mindfulness) for an average of 3.4 hours per week.

#### Procedure

Participants were recruited in two ways. First, psychology students from a mid-sized university in Australia were given course credit for completing the questionnaire (*n* = 363). Second, participants were sourced by a snowball method via a social media website where a brief description of the study was posted with a link to the online questionnaire (*n* = 82). This method has been used in similar scale development papers on self-compassion (Raes et al., 2011) and follows previous studies (Brown and Ryan, 2003; Sahdra et al, 2010) which have utilized community samples when developing measures to assess Buddhist psychological constructs.

All participants completed an online questionnaire containing the 64 items in their own time. Before being presented with the items, participants were prompted with the statement “Below are a number of statements related to your experiences and how you view yourself. Please read each item carefully and rate the extent to which you agree with each statement. Please answer according to what reflects your experience rather than what you think your experience should be.” All items were rated on a scale from 1 (Strongly Disagree) to 7 (Strongly Agree). All participants were presented with a consent information statement and provided their consent to participate by completing the questionnaire. Ethical approval for the study was granted by the Swinburne University Human Research Ethics Committee.

### Results

An exploratory factor analysis (EFA) was conducted on the 64 items (see Supplementary file) to determine the underlying factor structure of the items. The Keiser-Meyer-Olkin (KMO) measure of sampling adequacy was 0.93 and Bartlett's test for sphericity was significant (*p* < 0.001) indicating the data were appropriate for analysis (Tabachnick and Fidell, 1996). Factors were extracted with the principal-axis method of estimation, to provide the best chance of detecting factors if they exist (DeWinter and Dodou, 2012), and an oblique rotation was used as any factors were expected to be correlated. Based on the sample size, a factor loading cut-off of 0.30 was selected in accordance with the recommendation of Hair et al. (1998).

Using Kaiser's criterion (i.e., Eigenvalues above 1), one clear factor was identified explaining considerably more than each of the other factors (24.9%). The next closest factor identified explained 7.2% of the variance in the items. However, 20 items did not load on the first factor or cross-loaded on multiple factors. These items were removed from further analyses. Another 32 items (mostly negatively worded) were removed as they cross-loaded on multiple factors.

A second EFA was conducted to examine the new 16-item scale. Again, one factor explained considerably more variance than the others, however, seven items did not fall cleanly on the single factor. It was noted that items that displayed a specific emotional valence (e.g., “I worry about the *negative* thoughts I have about myself,” “I consciously try to only focus on the *positive* aspects of myself.”) tended not to load on a single factor. The decision was made to remove cross-loading items and two further items that had factor loadings <0.30.

A final EFA was conducted using only the seven items. EFA revealed a single factor that accounted for 44.63% of variance in the items. Factor loadings for these items are shown in Table 1. Furthermore, the items were internally consistent (α = 0.84). Alpha-if-item deleted results also indicated that the overall Cronbach's alpha coefficient would not increase if any items were removed. From this point on the seven items were referred to as the nonattachment to self (NTS) scale.

**Table 1:** Means, standard deviations, alpha-if-items-deleted and factor loadings of items for the nonattachment to self scale.

**Item**	***M***	***SD***	***FL***	***AID***
1. I can let go of unhelpful thoughts about myself.	4.63	1.54	0.72	0.81
2. I can let go of the need to control my life.	3.91	1.67	0.72	0.83
3. I don't get too caught up in the thoughts I have about myself.	4.11	1.51	0.71	0.81
4. As time goes on I feel less and less defined by the thoughts I have about who I am.	4.23	1.43	0.54	0.83
5. As time goes on I feel less and less of a need to be a certain way.	4.85	1.51	0.52	0.83
6. I can experience my personal ups and downs without getting caught up in them.	4.46	1.63	0.79	0.80
7. I can observe the positive and negative thoughts I have about myself without engaging in them.	4.32	1.50	0.80	0.80

*N = 445, M, mean; SD, standard deviation; FL, factor loading; AID, alpha if item deleted*.

## Study 2: Confirmatory Factor Analysis and Validity Assessment

The purpose of Study 2 was to replicate the 7-item factor structure and internal reliability of the NTS scale through two separate confirmatory factor analyses (CFAs) on two new samples. Study 2 also sought to establish the test-retest reliability of the new scale and examine criterion, convergent and discriminant validity of the NTS scale. As NTS is expected to be a relatively stable quality, scores on the scale were expected to be consistent over time. Further, as a dissolution of self-focus often occurs within the meditation process (Berman and Stevens, 2015), criterion validity was tested by comparing levels of NTS for those who engaged in contemplative practice relative to those who do not. It was expected that those who engaged with a contemplative practice would have higher levels of NTS than those who did not. The number of hours spent in contemplative practice was also expected to be positively related to NTS.

To establish convergent validity, the new scale was expected to correlate with the conceptually similar constructs of; nonattachment, mindfulness, and self-compassion. NTS was also expected to correlate with measures of psychological functioning; emotional stability, reduced rumination, self-transcendence, wisdom, and self-actualization. Further, as an over self-focus has been shown to be related to negative psychological outcomes (Mor and Winquist, 2002; Kyrios et al., 2016), negative correlations were expected between NTS scores and symptoms of depression, anxiety and stress, and positive correlations were expected with life satisfaction and psychological well-being. As nonattachment does not represent a detached state and requires presence and self-reflectiveness (Sahdra et al., 2015), to determine discriminant validity, weak-to-non-significant correlations were expected with measures of detachment; dissociation, depersonalization, absorption, amnesia, and lack of self-awareness. In addition, to ensure its distinctiveness from conceptually similar constructs; nonattachment and self-compassion, discriminant validity was tested with CFA, using nested models (Bagozzi et al., 1991), and when comparing unique variance explained in well-being variables.

### Participants and Procedure

Two separate samples were used for the two confirmatory factor analyses (CFAs). Participants in Sample 1 and Sample 2 were first-year psychology students from a mid-sized Australian university that received course credit for participation. All respondents completed an online survey at a time and place of their choosing. All respondents were presented with a consent information statement and provided their consent to participate by completing the questionnaire. Ethical approval for the study was granted by the Swinburne University Human Research Ethics Committee. As these two samples were obtained after Study 1, further demographic data was collected not previously collected in Study 1.

Sample 1 comprised 388 respondents (71 men & 317 women) aged from 18 to 77 (*M* = 35.33, *SD* = 10.80). Eighty percent of participants were born in Australia or New Zealand, 4.4% in the UK, 1.3% from India, 1.3% South Africa 1% from Iran, 1% from Malaysia, and 11% Other. Most respondents did not state any religious or spiritual affiliation (64.2%) or identified as Christian (21.9%); 5.4% identified with a general, non-religious spirituality, while 2.6% identified with a contemplative tradition (i.e., Buddhism, Vedanta), 2.3% identified as Muslim, 1% identified as Hindu, and 2.8% other.

Sample 2 comprised 338 respondents (76 men & 262 women) ranging from 18 to 75 years (*M* = 34.43 *SD* = 11.60). The respondents predominantly identified as Anglo-European (82.6%), followed by Asian (7%), Indian and sub-continent (2.6%), Middle Eastern (2%), African (1.7%), New Zealander or Pacific Islander (1.7%), or other (2.2%). Most respondents did not state any religious or spiritual affiliation (51.2%) or identified as Christian (24.4%); 13.1% identified with a general, non-religious spirituality, while 5.2% identified with a contemplative tradition (i.e., Buddhism, Vedanta), 1.7% identified as Muslim, 1.2% identified as Hindu, and 3.1% other.

### Measures

In addition to the new 7-item NTS scale developed in Study 1, a range of other measures were included to establish validity of the new scale. The measures included for this purpose are established measures of the constructs with strong reliability and validity statistics.

#### Criterion Validity

##### Meditation experience

Participants from sample 1 and sample 2 were asked: “Do you engage with a meditative or contemplative practice?” Participants were also asked to: “Please provide the approximate amount of hours you spend engaged in meditative/contemplative practice per week.”

#### Convergent Validity

##### Nonattachment

Nonattachment was assessed using a 7-item version of the original nonattachment scale (NAS-7; Elphinstone et al., 2015; Sahdra et al., 2016). The NAS-7 was drawn from the larger 30-item nonattachment scale (NAS; Sahdra et al, 2010) and has shown to have good reliability and validity when compared to the original NAS. Participants rated their agreement with 7 items (e.g., “I can let go of regrets and feelings of dissatisfaction about the past”) using a 7-point Likert scale from 1 (Strongly Disagree) to 7 (Strongly Agree).

##### Mindfulness

A 20-item short form of the Five Factor Mindfulness Questionnaire FFMQ (Tran et al., 2014) was used. The FFMQ consists of 20-items (e.g., “I am easily distracted,” “In difficult situations, I can pause without immediately reacting”). Capturing five factors of mindfulness, *observing, describing, awareness, non-judgment*, and *non-reactivity*, which are summed to provide an overall score of mindfulness. Items are rated on a 5-item Likert scale from 1 (Never, or very rarely true) to 5 (very often, or always true).

##### Self-compassion

The Self-Compassion Scale- Short Form (SCS-SF; Raes et al., 2011) is a 12-item measure drawn from the original 26 item self-compassion scale (SCS; Neff, 2003) designed to “measure self-compassion from the perspective of Buddhist Psychology” (Neff, 2003, p. 226). The 12-item scale has shown near perfect correlation (*r* = 0.97) with the larger SCS when measuring the single factor of self-compassion All items (e.g., “When something upsets me I try to keep my emotions in balance”) are rated on a Likert scale capturing the frequency of experiences from 1 (almost never) to 5 (almost always).

##### Emotional stability

The Emotional Reactivity Scale (ERS; Nock et al., 2008) assesses emotional sensitivity, emotional intensity, and emotional persistence across 21 items (e.g., “I get angry at people very easily”) rated on a 5- point Likert scale ranging from 0 (not at all like me) to 4 (completely like me). Scores were reversed and summed to give a total out of 84 with higher scores indicating higher levels of emotional stability.

##### Rumination

The rumination scale (Treynor et al., 2003) consists of 10 items, designed to measure repetitive thoughts about negative feelings, and their associated meanings. The scale is an adapted short form of the original Ruminative Response Scale (RRS; Nolen-Hoeksema and Morrow, 1991) to improve its construct validity. The frequency of items (e.g., “analyse recent events to try to understand why you are depressed”) are rated on a 4-point scale from 1 (almost never) to 4 (almost always).

##### Self-transcendence

The self-transcendence subscale from adult self-transcendence inventory (ATSI; Levenson et al., 2005) is an 9-item measure of self-transcendence with items (e.g., “I do not become angry as easily”) rated on a Likert scale from 1 (Disagree Strongly) to 4 (Agree Strongly). The ATSI is a well-established measure of self-transcendence when assessing the construct as a process of adult development.

##### Wisdom

The 12-Item Three-Dimensional Wisdom Scale (3D-WS-12; Thomas et al., 2017) was used to capture the dimension of wisdom. The 3D-WS-12 is a recently developed abbreviated version of the larger three-dimensional wisdom scale (3D-WS; Ardelt, 2003) and shows good reliability and validity when measuring a higher order single factor of wisdom (Thomas et al., 2017). Items (“When I am confused by a problem, one of the first things I do is survey the situation and consider all the relevant pieces of information”) are rated on a scale from 1 (Strongly Disagree) to 5 (Strongly Agree).

##### Self-actualization

The Short Index of Self-actualization (SISA; Jones and Crandall, 1986) measured self-actualization characterized as a process of maximizing full potential. The scale consisted of 15 items (e.g., “I do not feel ashamed of any of my emotions”) rated on a four-point scale from 1 (Disagree) to 4 (Agree), with higher scores representing greater amount of self-actualization.

##### Depression, anxiety, and stress

The 21-item Depression, Anxiety, and Stress Scale (DASS-21; Lovibond and Lovibond, 1995) was used to measure depression, anxiety and stress. The DASS−21 comprises three subscales of 7 items each capturing symptoms of depression (e.g., “I felt that life was meaningless”), anxiety (e.g., “I felt scared without any good reason”), and stress (e.g., “I felt I found it difficult to relax”). Respondents rate the extent to which they have experienced symptoms over the previous week on a scale ranging from 1 (“Did not apply to me at all”) to 4 (“Applied to me very much, or most of the time”).

##### Psychological well-being

Psychological well-being was measured by a 30-item version of the Psychological Well-being (PWB) Scale (Ryff, 1989; Ryff and Keyes, 1995). The PWB scale yielded a total score by summing the 30 items as well as individual scores for the six dimensions of *Autonomy, Purpose in Life, Environmental Mastery, Positive Relationships with Others, Personal Growth*, and *Self-Acceptance*, consisting of 5 items each. All items (e.g., “I like most aspects of my personality”) are rated on a 6-point scale from 1 (Strongly Disagree) to 6 (Strongly Agree).

##### Life satisfaction

Life satisfaction was measured with the Satisfaction with Life Scale (SWLS; Diener et al., 1985). The SWLS consists of five items (e.g., “in most ways my life is close to ideal”) rated on a 7-point scale from 1 (Strongly Disagree) to 7 (Strongly Agree) for scale totals ranging from 5 to 35.

#### Discriminant Validity

##### Dissociation

The Curious Experiences Survey (CES; Goldberg, 1999) is a 31-item measure amended from the Dissociative Experiences Scale (Bernstein and Putnam, 1986) to be more concise and easily understood. The CES assesses three factors of dissociation: depersonalization (e.g., “Had the experience of feeling that my body did not belong to me.”), amnesia (e.g., “Found evidence that I had done things that I do not remember doing.”), and absorption (e.g., “Found that I became so involved in a fantasy or daydream that it felt like it was really happening to me.”). Items are rated on a 5-point scale from 1 (This never happens to me) to 5 (This almost always happens to me).

##### Self-awareness

The Situational Self-Awareness Scale (SSAS; Govern and Marsch, 2001) is a 9-item measure of self-awareness. The SSAS measures 3 subscales capturing private self-awareness or internal state awareness (e.g., “Right now, I am aware of my innermost thoughts.”), public self-awareness (e.g., “I am concerned what other people think of me.”), and awareness of immediate surroundings (e.g., “Right now, I am keenly aware of everything in my environment”). Item are measured on 7-point scale from 1 (Totally Disagree) to 7 (Totally Agree).

## Results

### Confirmatory Factor Analysis

**Figure 1:**
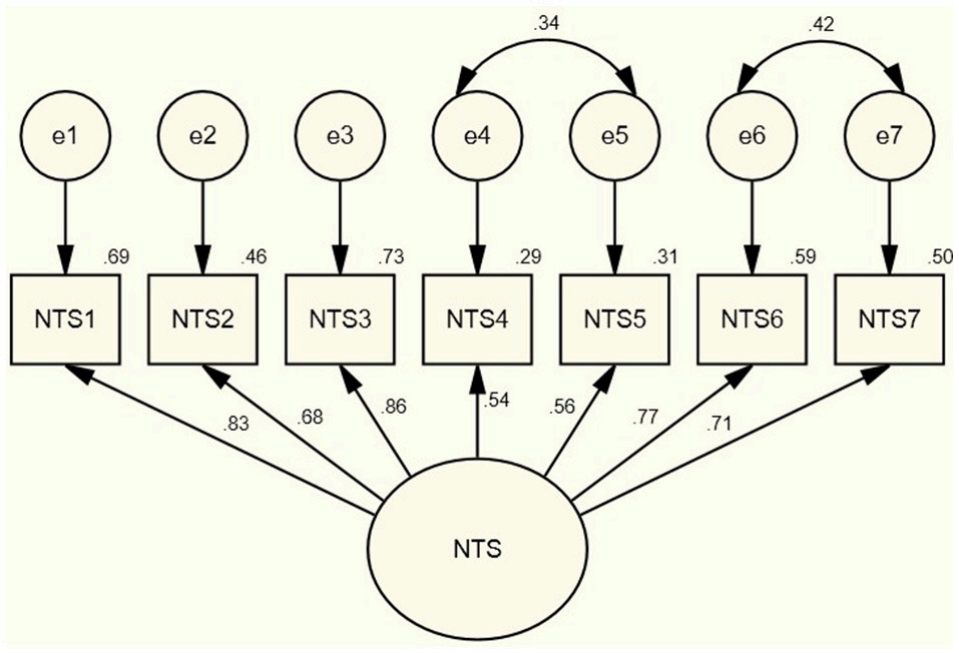
Confirmatory factor analysis using structural equation model for the 7-item nonattachment to self scale for sample 1.

An initial CFA using a structural equation model (SEM) was conducted to test the model fit for Sample 1. The initial model fit fell outside accepted criteria (CFI = 0.92, TLI = 0.88, RMSEA = 0.14, and SRMR = 0.06). Examination of the modification indices in the model revealed covariances between items 4 and 5 and items 6 and 7. Examination of the content of these items (See Table 1) revealed they were semantically similar but addressing subtly different aspects of self. Items 4 and 7 appear to directly capture how individuals interact with their thoughts about self, while items 5 and 6 capture aspects of the self in experience. As these items were determined to be semantically similar but importantly distinct constructs, a CFA with covariance parameters was conducted on Sample 1 (See Figure 1). This analysis revealed a good fit with the data in Sample 1 [$\chi_{\left(11\right)}^{2}$ = 22.94, *p* = 0.02, CFI = 0.99, TLI = 0.98, RMSEA = 0.05, SRMR = 0.02]. The one factor solution was confirmed with seven items falling on a single factor an explaining 54.37% of the variance in the items. The internal consistency of these items was excellent with a Cronbach's coefficient alpha of 0.88.

**Figure 2:**
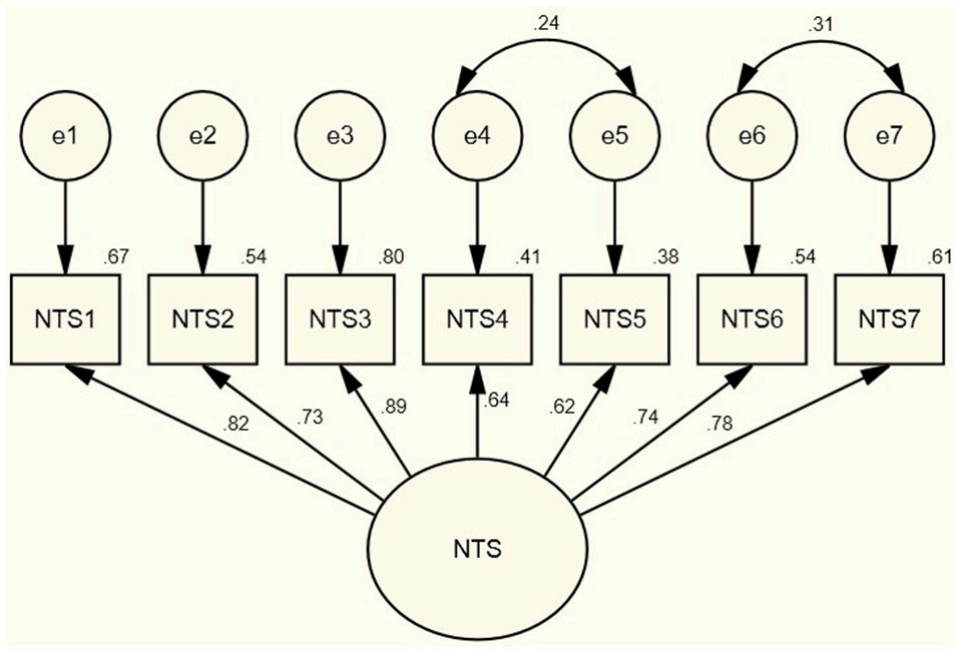
Confirmatory factor analysis using structural equation model for the 7-item nonattachment to self scale for sample 2.

To confirm and replicate the factor structure of the CFA for sample 1, a second CFA with covariance parameters was conducted on Sample 2 (see Figure 2). This analysis revealed an adequate model fit with the data [$\chi_{\left(11\right)}^{2}$ = 23.90, *p* = 0.01, CFI = 0.98, TLI = 0.97, RMSEA = 0.08., SRMR = 0.02). Further confirming the factor structure, the single factor solution explained 60.3% of the variance in the items and Cronbach's coefficient alpha was again excellent at 0.91.

Potential gender differences were also explored with *t*-tests in both samples. Results showed no significant difference in NTS between men (Sample 1 *M* = 31.68, *SD* = 8.74, *n* = 74; Sample 2 *M* = 31.96, *SD* = 7.02, *n* = 76) and women (Sample 1 *M* = 31.25, *SD* = 8.88, *n* = 262; Sample 2 *M* = 31.55, *SD* = 8.52, *n* = 317) in Sample 1 [*t*_(386)_ = 0.374, *p* = 0.709) or Sample 2 [*t*_(334)_ = 0.364, *p* = 0.716).

### Test-Retest Reliability

Test-re-test reliability was obtained from a sub-sample of 29 participants who originally completed the scale in Study 1, who consented to complete the NTS scale at a later date. The modal time between completions of the NTS scale was 36 days. Respondents' scores at both timepoints were highly correlated (*r* = 0.80, *p* < 0.001) indicating that scores on the NTS scale are consistent over time.

### Criterion Validity

To test the criterion validity, levels of NTS were compared between participants who did and did not engage in contemplative practice. Independent samples *t*-tests in both samples revealed NTS scores for respondents engaging in contemplative practice (Sample 1, *M* = 33.34, *SD* = 8.04, *n* = 163; Sample 2, *M* = 32.93, *SD* = 8.81, *n* = 173) were higher than respondents who did not (Sample 1, *M* = 30.39, *SD* = 8.21, *n* = 225; Sample 2, *M* = 29.72, *SD* = 0.8.60, *n* = 166). This difference was significant in in both samples [Sample 1 *t*_(386)_ = 3.53, *p* < 0.001; *Cohen's d* = 0.35, Sample 2 *t*_(337)_ = 3.42, *p* = 0.001; *Cohen's d* = 0.48]. NTS scores also showed a weak positive correlation with hours spent in contemplative practice per week: Sample 1, *r* = 0.10, *p* = 0.04; Sample 2, *r* = 0.23, *p* < 0.001).

## Convergent and Discriminant Validity

### Convergent Validity

Correlations for the convergent validity measures (See Table 2) indicate the NTS scale showed weak-to-moderate to moderate-to-strong correlations with each of the convergent measures (*r* = −0.34 to *r* = 0.72). Results showed a moderate-to-strong positive relationship between NTS and the theoretically aligned constructs of nonattachment and self-compassion, and moderate positive relationship between NTS and mindfulness. NTS also showed weak-to-moderate negative correlations to emotional stability and rumination, and moderate positive correlations with self-transcendence, self-actualization and wisdom.

**Table 2:** Internal reliabilities coefficients and correlations of nonattachment to self to convergent validity measures.

	**Sample 1 *n* = 388**	**Sample 2 *n* = 338**	***Alpha*** **Samples**	
			**1**	**2**
**CONVERGENT VALIDITY**				
Nonattachment	0.71^**^		0.95	
Mindfulness	0.56^**^		0.91	
Self-Compassion	0.72^**^		0.95	
Emotional Stability	0.55^**^		0.96	
Rumination	−0.34^**^		0.84	
Self-Transcendence		0.56^**^		0.87
Self-actualization		0.54^**^		0.73
Wisdom		0.41^**^		0.76
**PWB**	0.67^**^		0.92	
Environmental Mastery	0.60^**^		0.81	
Personal Growth	0.32^**^		0.72	
Autonomy	0.45^**^		0.75	
Self-acceptance	0.63^**^		0.86	
Life Purpose	0.56^**^		0.61	
Relationships	0.47^**^		0.81	
Life satisfaction		0.46^**^		0.89
**DASS**				
Depression	−0.51^**^		0.91	
Anxiety	−0.46^**^		0.89	
Stress	−0.52^**^		0.88	

** *p < 0.001*.

Correlations for the well-being variables (See Table 2) were all in the expected direction with the NTS scale showing weak to moderate (*r* = 0.25 to *r* = 0.67) relationships to all the well-being measures. Specifically, the NTS scale displayed weak-to-moderate positive correlations with all facets of PWB and life satisfaction, and displayed moderate negative correlations with symptoms of depression, anxiety, stress. However, the internal reliability of the subscale “life purpose” was below acceptable. As the scale was short and represented a subscale of the PWB scale, the decision was made to proceed with the analysis.

### Discriminant Validity

Correlations for discriminant validity (See Table 3) were either non-significant or weak and fell within expected parameters (*r* ranged from 0.06 to −0.39). Specifically, the NTS scale was not significantly related to measures of amnesia, absorption or total situational self-awareness, and only showed a weak negative relationship to dissociation, depersonalization, private self-awareness, public self-awareness, and environmental self-awareness.

**Table 3:** Internal reliabilities coefficients and correlations of nonattachment to self to discriminant validity measures.

**Discriminant Validity**	**Sample 2**	**Alpha**
**Dissociation (total)**	−0.11^*^	0.96
Amnesia	−0.08	0.81
Absorption	−0.07	0.85
Depersonalization	−0.11^*^	0.91
**Situational Self-awareness (total)**	−0.04	0.75
Self in Surroundings	0.25^**^	0.85
Private Self-awareness	0.18^*^	0.73
Public Self-awareness	−0.39^**^	0.81

*N = 338*.

** *p < 0.001*,

* *p < 0.05*.

### Distinctiveness From Nonattachment and Self-Compassion

Due the strength of the correlation between NTS and self-compassion, the decision made to test the distinctiveness of NTS from general nonattachment *and* self-compassion. To test the distinctiveness of NTS from general nonattachment and self-compassion, two separate CFAs were conducted using nested models (see Bagozzi et al., 1991). Using nested models to test discriminant validity involves comparing the fit of two models, an unconstrained model and a constrained model. The original (unconstrained) model, where the relationship between two conceptually similar latent variables are allowed to covary, is compared with a nested (constrained) model where the correlation between the latent variables is set to 1, indicating that both constructs are identical (Schweizer, 2014; Shaffer et al., 2016). The fit of the constrained and unconstrained models are then compared using a chi-square difference test, and comparing the difference in comparative fit index (CFI) and root mean square error of approximation (RMSEA). If the constrained model shows a significantly worse fit than the unconstrained model, then discriminant validity is supported. Nested models are a rigorous and widely-accepted SEM-based approach to discriminant validity (Shaffer et al., 2016).

In the present study, two separate nested models were conducted on sample 1, to test discriminant validity. The first test compared NTS to nonattachment, using latent variables, with the constrained model setting the relationship between the NTS and nonattachment to 1 (indicating they are the same construct). Results showed the constrained model to be a worse fit than the unconstrained model (see Table 4). The second test compared NTS with self-compassion, using latent variables, with the constrained model setting the relationship between NTS and self-compassion at 1. Results showed the constrained model was a worse fit than the unconstrained model (see Table 4). Based on accepted criteria (ΔCFI ≥ 0.01, Cheung and Rensvold, 2002; ΔRMSEA ≥ 0.015, Chen, 2007), the results show a difference between the models, suggesting the distinctiveness of NTS from nonattachment and self-compassion.

**Table 4:** Fit indices comparing nested models to determine discriminant validity between nonattachment to self, and nonattachment and self-compassion.

	**χ^2^**	***df***	**CFI**	**RMSEA**	**Δ χ^2^**	**ΔCFI**	**ΔRMSEA**
**NTS AND NONATTACHMENT**							
Unconstrained	261.29	76	0.93	0.08			
Constrained	438.45	77	0.87	0.11	177.16^**^	0.07	0.03
**NTS AND SELF-COMPASSION**							
Unconstrained model	888.32	151	0.81	0.11			
Constrained Model	1151.21	152	0.74	0.13	262.90^**^	0.05	0.02

Sample 1, N = 388

** *p < 0.001*.

To further investigate how NTS it distinguished from nonattachment and self-compassion, four multiple regression analyses were conducted to determine whether NTS distinctly predicted well-being variables when measured alongside nonattachment, and a further four multiple regression analyses were conducted to determine whether NTS distinctly predicted well-being variables when measured alongside self-compassion. Table 5 reports the unstandardized and standardized regression coefficients, and standard errors for each multiple regression analysis. Results showed that when NTS was measured alongside nonattachment, NTS distinctly predicted of PWB, depression, anxiety, and stress. Analyses further revealed that, when NTS was measured alongside self-compassion, NTS distinctly predicted PWB, depression, anxiety, and stress.

**Table 5:** Regression coefficients and standard errors from multiple regression models comparing unique relationships of nonattachment to self, nonattachment and self-compassion, with psychological well-being, depression, anxiety, and stress.

	**PWB**			**Depression**			**Anxiety**			**Stress**		
	**B**	**SE**	**β**	**B**	**SE**	**β**	**B**	**SE**	**β**	**B**	**SE**	**β**
**DISCRIMINANT ANALYSIS 1**												
Nonattachment	1.64	0.16	0.34^**^	−0.12	0.04	0.20^*^	−0.13	0.03	0.21^*^	−0.13	0.04	0.19^*^
NTS	0.89	0.12	0.26^**^	−0.18	0.03	0.37^**^	−0.16	0.04	0.32^**^	−0.20	0.03	0.38^**^
**DISCRIMINANT ANALYSIS 2**												
Self-compassion	1.03	0.14	0.39^**^	−0.16	0.03	0.33^**^	−0.15	0.03	0.28^**^	−0.17	0.03	0.31^**^
NTS	1.01	0.14	0.38^**^	−0.13	0.03	0.25^**^	−0.12	0.03	0.23^**^	−0.15	0.03	0.27^**^

*Sample 1, N = 388*,

** *p < 0.001*,

* *p < 0.01, PWB, Psychological Wellbeing*.

## General Discussion

The aim of the research was to develop and validate a reliable measure of nonattachment to self (NTS). This resulted in the creation of a new 7-item measure of NTS loading on a single factor that was confirmed using two separate CFAs. The new scale shows good internal consistency, test-retest reliability, and criterion validity. The scale was also shown to have good convergent and discriminant validity and importantly, results indicate NTS is an empirically distinct construct from nonattachment and self-compassion. As expected, NTS related to measures of positive psychological functioning and well-being and did not represent a detached or dissociated state. The results suggest the NTS scale is valid, reliable over time, and distinct to nonattachment in general. Accepting any self-related feelings, thoughts or concepts, regardless of valence, and not forcibly try to change these to fit with an ideal, appears to be a way of relating the self that is related to positive psychological outcomes.

The validity process provided empirical support for the distinctiveness of NTS from general nonattachment. This was important as it supports the continued study of NTS as a separate construct. Distinguishing NTS from nonattachment indicates there are differences between how individuals attach to external experience and how they attach to their self-related experience. As the construct of the self is central to the many roles individuals play (Bhar and Kyrios, 2016; Shiah, 2016), taking a non-attached stance toward the self can affect many aspects of individuals' lives. In contrast, an individual may have attachments to external experience that may be specific (e.g., physical injury, interpersonal confrontation) but that may not necessarily affect other aspects of their life. Interestingly, results indicated one area NTS may be more beneficial than general nonattachment, is for ameliorating the impact of negative psychological symptoms; depression, anxiety, and stress. This may be due to the self-playing a central role is psychopathology (Kyrios et al., 2016) and an over self-focus being linked with negative mental health symptoms (Levenson et al., 2001; Mor and Winquist, 2002).

In addition to nonattachment, NTS was also shown to be distinct from self-compassion. This means that taking a non-attached stance toward the self differs from taking a balanced and compassionate stance toward negative emotions (Neff, 2008). This distinction points to the notion that reducing any self-fixation, regardless of valence, is different from reducing the impact of negative self-related experience. The findings also highlight that, in addition to the effects of being more self-compassionate, reducing fixation on the self, whether positive or negative, can positively impact an individual's well-being and reduce negative psychological symptoms.

The results for convergent validity also indicated that NTS was related to measures of positive psychological functioning. NTS was related to greater emotional stability and less ruminative thinking. Emotional stability refers to an individuals' capacity to be able to be balanced when responding to emotionally provoking stimuli (Hills and Argyle, 2001). The findings suggest that emotional reactivity to self-referent stimuli, such as negative self-evaluations or criticism from others, may be ameliorated by taking a more flexible approach to the self-concept and reducing the incongruence between stimuli and self-concept. Similarly, whereas rumination involves unintentional recurring thoughts with a positive or negative self-focus, that can perpetuate symptoms of depression (Krieger et al., 2013), NTS indicates a reduction in the positive or negative self-focus and a more flexible self-concept. This would assist in reducing obtrusive thoughts or letting them pass without having them reoccur. These findings support the theorized benefits of taking a more flexible stance toward the self-concept on the way individuals manage their emotions and cognitions.

In addition to adaptive functioning, NTS related to wisdom, self-actualization and self-transcendence. Wisdom, self-actualization and self-transcendence are taken as measures of advanced psychological development that indicate the higher stages of psychological growth (Whitehead et al. submitted). The present findings indicate that being flexible and non-attached in relation to the self may facilitate a transcendence of self-focus that is implicit in the later stages of psychological development (Cook-Greuter, 2000; Hartman and Zimberoff, 2008). Potentially, by removing fixation on the self and the need for self-related experience to be one way or other, individuals may be able to reduce the self-bias that can limit development of wisdom and self-transcendence (Whitehead et al. submitted). The present findings indicate that NTS can be associated with the growth process and supports the Buddhist notion that nonattachment to the self develops over time and is a goal that is worked toward (Donner, 2010). This is also supported by the observed relationship between contemplative practice and NTS and indicates NTS can develop over time, in conjunction with contemplative practice. Practices like meditation can assist in a dissolution of self-focus (Emavardhana and Tori, 1997; Berman and Stevens, 2015) and can create distancing from the immediacy of experience (Bishop et al., 2004; Neff, 2008), which can facilitate the letting go of attachment to thoughts, feelings and concepts about the self.

There are a number of implications of the current research. The development and validation of the NTS scale provides empirical support for a construct of NTS distinct from nonattachment and self-compassion. NTS appears to be a distinct quality that can make a positive unique contribution to individuals' mental health and psychological growth, beyond the more widely studied constructs of nonattachment and self-compassion. This research also provides insight into the possible benefits of understanding the self as a fluid rather than a static entity and invites research on the Buddhist notion of the self as a dynamic process. As individuals' notions of self play a central role in their well-being (Kyrios et al., 2016), understanding the self as a more dynamic process and taking a more non-attached stance toward the self-concept, rather than taking a positive stance toward the self, may be a fruitful area of study in relation to individuals' well-being and quality of life in general.

The findings also have implications for individuals whose self-related feelings make it difficult to have any positive self-interactions. Individuals whose negative psychological symptoms impact their ability to benefit from strategies such as self-compassion (Pauley and McPherson, 2010; Gilbert et al., 2011), may still be able to gain benefit from taking a more non-attached stance toward their self. As NTS does not require a positive interaction with self, it is not in opposition to feelings of low self-worth or hopelessness. It could therefore be met with less resistance than strategies that require a positive self-focus. Future research comparing NTS with constructs like self-compassion, investigating whether NTS acts as a protective factor against negative psychological symptoms, and whether specific interventions can target NTS could further elucidate the construct.

A number of methodological considerations are relevant to the present studies. As the samples were predominantly of university students and with considerably more women than men, the generalizability of the findings is limited. Nevertheless, the gender bias may not detract markedly from the findings as no gender differences in NTS were found. However, further research is needed on larger samples drawn from across the community to establish generalizability. Future research using stratified sampling may also assist in detecting variance in NTS in different areas such as culture and religion. Additionally, as this study did not use a clinical sample, the findings in relation to depression, anxiety and stress may not apply to individuals experiencing clinical levels of these symptoms and future work is needed to establish whether the relationships found also hold in a clinical population. Longitudinal studies on NTS are also needed to examine how NTS develops over time. Despite these limitations, the present study provides a robust development and validation process for the new measure of NTS that appears relevant to a range of areas associated with mental health and quality of life in general.

In conclusion, the present studies established the reliability and preliminary evidence of validity on a new measure of NTS. NTS emerged as a quality related, but distinct from other Buddhist psychological constructs, and that taking a more flexible, non-attached stance toward self-related thoughts, feelings and concepts can be beneficial for individuals' well-being and psychological functioning. The findings also indicate that NTS may provide unique benefit to individuals' well-being over and above the effects of other similar measures and may provide an avenue for healthy interaction with the self-concept for individuals that struggle with a positive self-focus.

## Author Contributions

RW responsible for original conception, data collection, data analysis, major contribution to writing manuscript. GB played a role in early conceptualization, study design, much drafting and re-drafting. BE assisted in data analysis and study design, assisted in drafting process. YY assisted at early conceptual stage, assisted in data collection. GM assisted in conceptual issues for publication, assisted in drafting process.

### Conflict of Interest Statement

The authors declare that the research was conducted in the absence of any commercial or financial relationships that could be construed as a potential conflict of interest.
