# Supplementary material for: Letting Go of Self: The Creation of the Nonattachment to Self Scale
Source: Front Psychol. 2018 Dec 13;9:2544. doi: 10.3389/fpsyg.2018.02544 (PMC6300706; doi:10.3389/fpsyg.2018.02544)
Supplement: Supplementary file 1 [file Table_1.DOCX]

Table 1.

*Item Pool of One Hundred and Sixteen Items for the Creation of the Nonattachment to Self Scale.*

|  | Item Pool |  |
| --- | --- | --- |
| 1. I can 'let go' of ideas I have about myself 2. I try to suppress negative thoughts I have about myself 3. I feel no need to protect or defend who I am. 4. At times, I can watch my self without having to attach to it. 5. I try and put a version of myself that I think people will like. 6. I rarely engage with thoughts I have about myself 7. My attitudes towards myself are very flexible 8. I worry a lot about what other people think of me 9. I remain calm when people challenge views that are important to me. 10. My self is only a collection of thoughts. 11. My life story defines me. 12. As time goes on I feel less and less defined by the story of my life. 13. I am no better or worse than other people 14. My concept of myself is something that is quite fixed 15. I rarely feel the need to defend my views 16. I invest a lot of time and energy into how I come across to others 17. I try not to take myself too seriously 18. I feel the need to build myself up to others 19. My personality defines who I am 20. I can remain present without engaging in the ups and downs of my life story 21. I cling to those aspects of myself I like 22. I can experience my personal ups and downs without getting caught up in them 23. I try not to hold on too tightly to my opinions and beliefs 24. I can observe the positive and negative thoughts I have about myself without engaging in them. 25. Building a strong sense of self is very important to me. 26. My personality is very important to me. 27. It is important to me that people like me 28. I spend a lot of time worrying about what people think of me 29. I don't worry about what other people think of me 30. I have experienced moments where my sense of being a separate self disappears 31. I don't get too caught up in thoughts I have about myself 32. I need to be a certain way to succeed in life. 33. Sometimes I feel as though I am part of something much bigger than myself. 34. I worry about myself a lot 35. I often think about parts of myself that I would like to change. 36. I feel uncomfortable when people tell me things about myself I don’t agree with. 37. I can let go of feelings of defensiveness 38. I am very flexible when it comes to defining myself. 39. As time goes on I feel less defined by the thoughts I have about who I am. | 1. I try not to hold fixed definitions of myself whether they be good or bad. 2. All experience is transient, including the experience of the self. 3. I am kind and accepting towards my self 4. I don't get hung up on wanting to be a perfect person. 5. I often judge myself harshly 6. I often seek praise from others. 7. At times I feel I am something more than an individual self. 8. I tend to dwell on aspects of my personality I don't like. 9. I consciously try to only focus on the positive aspects of myself. 10. It is important for me to feel I am right. 11. I try to let go of judgmental thoughts I have about myself. 12. There is nothing more important than developing my self. 13. My individuality is very important to me. 14. My thoughts and feelings about myself tend to affect me greatly 15. I am aware my thoughts are aimed at making me feel good or bad 16. I actively try to think positively about myself. 17. I spend a lot of time working on developing a strong sense of self 18. I have experienced moments when the individual self has dropped away. 19. I rarely dwell on thoughts I have about myself. 20. I try not to get caught up in the ups and downs of my life story 21. I try to stay out of the affairs of others 22. When certain aspects of my self are challenged I become defensive 23. I tend to fixate on thoughts I have about myself 24. I tend to think a lot about my appearance. 25. I feel secure and grounded in my life 26. I often feel tense about my life situation 27. I am accepting of myself when things don't work out 28. I often worry when I don't meet my own expectations 29. I feel strongly invested in the way my life turns out 30. I often compare myself to others 31. As life goes on, I worry about myself less and less 32. I tend to hold on tightly to good aspects of myself and reject bad things about myself 33. I rarely worry what other people think of me. 34. I often get angry with other people 35. I am accepting of people who are different to me 36. It is very important for me to achieve specific goals 37. I can let go of the need to control my life 38. I feel that surrendering to life provides a sense of freedom 39. I am often influenced by other people's expectations of me | 1. I generally remain steady throughout the ups and downs of life. 2. I can consciously surrender to the ups and downs of life. 3. My actions are usually motivated by others 4. I make a conscious effort not to engage with judgements about myself 5. As time goes on, I feel less of a need to be a certain way 6. When I do a favour for someone, I expect the favour to be returned. 7. I feel like I am controlled by my what my ego wants. 8. My wants and needs govern my life 9. I can let go of unhelpful thoughts about myself. 10. I rarely engage with negative self-talk 11. I wish I was a better person 12. I can observe myself from an external perspective. 13. I am very critical of myself 14. I am aware that my thoughts are often aimed at building me up or putting me down 15. I can admit my shortcomings without shame or embarrassment 16. I invest a lot in my self-image. 17. I am aware that trying to control my thoughts is futile 18. My sense of personal identity is very important to me 19. I can let go of judgements I have about myself 20. Thoughts I have about myself rarely worry me 21. I am accepting of negative thoughts about myself. 22. I often focus on how I can be a better person 23. I actively try to be vulnerable with others 24. I am very interested in finding out the answer to the question "who am I?" 25. I often rate myself as better or worse than others. 26. I can watch my thoughts without getting caught up in them 27. I am aware that my beliefs and opinions are biased. 28. I feel I am equal to all other people. 29. I can smile when I realise how I sometimes make life difficult for myself. 30. Realising the truth of who I am is the most important aspect of my life 31. I have had moments of great 32. joy in which I suddenly had a clear, deep feeling of oneness with all that exists 33. I need to be a certain way to be happy 34. As time goes on I have become less identified with my individual concerns. 35. Who I am cannot be defined. 36. Notions of "good" and "bad" are just thoughts 37. There is no objective "right" or "wrong" 38. As time goes on, I have become less self-centred. |

Table 2.

*Factor Loadings from Exploratory Factor Analysis on Sixty-Four Items of the Nonattachment to Self Scale*

| Items | 1 | 2 | 3 | 4 | 5 | 6 |
| --- | --- | --- | --- | --- | --- | --- |
| I feel no need to protect or defend who I am. | .531 |  |  |  |  |  |
| As time goes on I have become less self-centered. | .432 |  |  |  |  |  |
| I can let go of unhelpful thoughts about myself. | .678 |  |  |  |  |  |
| I can observe myself from an external perspective. | .475 |  |  |  |  |  |
| I wish I was a better person. | .483 |  |  |  |  |  |
| I can admit my shortcomings without shame or embarrassment. | .472 | -.376 |  |  |  |  |
| I smile when I realise how I sometimes make life difficult for myself. | .347 |  |  |  |  |  |
| I am aware that my beliefs and opinions can be biased. |  |  |  |  |  |  |
| I am rarely worried by thoughts I have about myself. | .637 |  |  |  |  |  |
| I find it hard to define myself. |  |  |  |  |  |  |
| At times, I can see that my 'self' is just a thought. |  |  | .466 |  |  |  |
| I invest a lot in my self-image. | .395 |  |  |  |  |  |
| I am very critical of myself. | .618 |  |  |  |  |  |
| It is important for me to feel I am right. | .470 |  |  |  |  |  |
| I rarely engage with negative self-talk. | .610 |  |  |  |  |  |
| I can let go of the need to control my life. | .562 |  |  |  |  |  |
| As life goes on, I worry less and less about myself. | .635 |  |  |  |  |  |
| I often rate myself as better or worse than others. | .506 |  |  |  |  |  |
| I feel strongly invested in the way my life turns out. |  | .579 |  |  |  |  |
| I am accepting of myself when things don't work out. | .697 |  |  |  |  |  |
| I invest a lot of time and energy into how I come across to others. | .503 | .329 |  |  |  |  |
| I rarely dwell on thoughts I have about myself. | .605 |  |  |  |  |  |
| I can let go of feelings of defensiveness. | .671 |  |  |  |  |  |
| My self is only a collection of thoughts. |  |  | .369 |  |  |  |
| As time goes on I feel less and less defined by the story of my life. | .412 |  | .367 |  |  |  |
| I feel that I am equal to all other people. | .583 |  |  |  |  |  |
| I try not to take myself too seriously. | .559 |  |  |  |  |  |
| I rarely feel the need to defend my views. | .477 |  |  |  | -.360 |  |
| I feel the need to build myself up to others. | .439 |  |  | .410 |  |  |
| I try to suppress negative thoughts I have about myself. |  | .364 |  |  |  |  |
| I feel strongly invested in the way my life turns out. |  | .550 |  |  |  |  |
| My personality defines who I am. |  | .390 |  |  |  |  |
| I try not to hold on too tightly to my opinions and beliefs. | .392 |  | .332 |  |  |  |
| I rarely worry about what other people what people think of me. | .622 |  |  |  |  |  |
| I don't get too caught up in the thoughts I have about myself. | .709 |  |  |  |  |  |
| I often think about parts of myself that I would like to change. | .411 | .353 |  |  |  |  |
| I feel uncomfortable when people tell me things about myself I don't agree with. | .410 |  |  |  |  |  |
| I am very flexible when it comes to defining myself. | .512 |  |  |  |  |  |
| My life story defines me. | .324 |  |  |  |  |  |
| As time goes on I feel less and less defined by the thoughts I have about who I am. | .501 |  |  |  |  |  |
| I try not to hold fixed definitions of myself whether they be good or bad. | .555 |  |  |  |  |  |
| I don't get hung up on wanting to be a perfect person. | .688 |  |  |  |  |  |
| I often judge myself harshly. | .584 |  |  |  |  |  |
| I often seek praise from others. | .547 |  |  |  |  |  |
| I tend to dwell on those aspects of myself I don't like. | .654 |  |  |  |  |  |
| I try to let go of judgmental thoughts I have about myself. | .505 |  |  |  |  |  |
| As time goes on I feel less and less of a need to be a certain way. | .563 |  |  |  |  |  |
| I often dwell on experiences that make me judge myself harshly | .674 |  |  |  |  |  |
| My thoughts and feelings about myself tend to affect me greatly. | .642 |  |  |  |  |  |
| My individuality is very important to me. |  | .474 |  |  |  |  |
| I consciously try to only focus on the positive aspects of myself. |  | .452 |  |  |  |  |
| I worry about the negative thoughts I have about myself. | .661 |  | -.326 |  |  |  |
| I tend to fixate on thoughts I have about myself. | .661 |  |  |  |  |  |
| I fixate on those aspects of myself I like. |  | .453 |  | .444 |  |  |
| I need to be a certain way to be happy. | .579 |  |  |  |  |  |
| I cling to those aspects of myself I like. | .335 | .444 |  | .374 |  |  |
| I can experience my personal ups and downs without getting caught up in them. | .738 |  |  |  |  |  |
| My sense of personal identity is very important to me. |  | .542 | .375 |  |  |  |
| My self-image has become less important in my life. | .540 |  |  |  |  |  |
| I respond to praise and criticism similarly. |  |  |  |  |  |  |
| My reputation is very important to me. | .361 | .334 |  |  |  |  |
| As time goes on I have become less identified with my personal failures. | .570 |  |  |  |  |  |
| As time goes on my individuality has become less important. |  |  | .479 | -.352 |  |  |
| I can observe the positive and negative thoughts I have about myself without engaging in them. | .716 |  |  |  |  |  |

*Note:* *Factor shown for all Eigenvalues above 1*. *Factor loadings below .30 not shown*.
